# Adult Avoidant Attachment, Attention Bias, and Emotional Regulation Patterns: An Eye-Tracking Study

**DOI:** 10.3390/bs13010011

**Published:** 2022-12-23

**Authors:** Arcangelo Uccula, Beniamina Mercante, Lavinia Barone, Paolo Enrico

**Affiliations:** 1 Department of History, Human Sciences and Education, University of Sassari, 07100 Sassari, Italy; 2 Department of Biomedical Sciences, University of Sassari, 07100 Sassari, Italy; 3 Department of Brain and Behavioral Sciences, University of Pavia, 27100 Pavia, Italy

**Keywords:** avoidant attachment strategies, lifespan development, emotional development, eye-tracker, attachment representation, eye movement

## Abstract

Proximity-seeking in distress situations is one of attachment theory’s primary strategies; insecure individuals often also develop secondary strategies. The mechanisms implied in attachment deactivation constitute a key issue in the current debate related to their role in support-seeking. The main aim of this study is to investigate the attachment deactivation strategy and the processes of proximity/support-seeking under distress conditions by analyzing the attentional processes (i.e., an essential emotion-regulation strategy), using eye-tracking techniques. Seventy-two participants (45 female; M_age_ 23.9 ± 3.97) responded to the ECR-R questionnaire in order to identify their attachment style. They participated in an experimental situation in which they had to choose between pictures of care or pictures of food, following the presentation of threatening or neutral prime conditions (via the pictures’ stimuli). Results showed that a care–consistency response pattern was the most frequent pattern of response, particularly under a threatening condition; on the contrary, only avoidant individuals showed a lower care–consistency response pattern by choosing food pictures. The overall findings demonstrate that avoidant individuals used the deactivation strategy to process comfort-related attachment pictures, suggesting that they considered these stimuli to be threatening. The implications for attachment theory and particularly for avoidant strategies are discussed.

## 1. Introduction

According to attachment theory, the biologically rooted, innate attachment behavioral system motivates humans to seek the closeness of significant others in times of threat and distress [1,2]. Individual attachment strategy differences in proximity-seeking depend on the quality of interactions with an attachment figure during childhood, which may affect how a person perceives and processes emotional content [3]. Primary dyadic experiences of emotional regulation are internalized over time, resulting in internal working models (IWMs) of attachment [4]. These models are organized as scripts and conceptualized as cognitive–affective structures that are executed automatically when processing new information [5]. Individual differences have been observed in IWMs that can be assigned to three prototypical patterns [6]. Securely attached individuals have an IWM of their caregiver as helpful and responsive to their emotional needs. When the caregiver is unavailable, individuals learn secondary strategies [7]. In adults, these strategies have been conceptualized into attachment anxiety and avoidance. Anxiety is characterized by hyper-activating strategies to maintain closeness with the caregiver, while avoidance is characterized by the deactivation of the attachment system.

Understanding the processes implied in avoidant attachment plays a central role in attachment theory [8]. The process of deactivation of avoidant individuals has received particular interest among researchers because of the involvement of specific attentional processes [9]. It has been proposed that one way of regulating negative affect is to limit attention to potentially threatening material, a process termed “defensive exclusion” by Bowlby [9]. Later studies suggested that an individual’s emotional processing of avoidant attachment is closely related to their attention bias [10]. Specifically, avoidant individuals tend to divert attention to negative and positive attachment-related information (e.g., emotional material such as words, pictures, emotional faces, and issues related to closeness and separation) [9]. Avoidant individuals have negative representations of attachment figures, which are considered threatening when closeness and support are needed [11]. In fact, a shift of attention away from negative and positive images of the mother during childhood [12] and negative and positive stimuli during adulthood [13] have been demonstrated. It seems plausible, thus, that caring images may be considered to be threatening stimuli by avoidant individuals and treated as such [14]. Szymanska [15] also found a faster autonomic response to distress pictures and shorter fixation times on both distress and comfort images. The research shows how [10] the deactivation of attention to arousing stimuli is consistent with the avoidance of attachment-related cues by avoidant individuals. However, inconsistent findings have been collected concerning key features of avoidant attentional strategies thus far [16]. In fact, while the relevant research shows a diversion of attention from attachment-related stimuli [10], a greater perceptual vigilance to emotional stimuli has been also demonstrated, indicating lower perceptual thresholds for emotional stimuli [17,18,19]. Indeed, avoidant individuals appear to be strongly engaged in perceptual vigilance for visual–emotional stimuli, with emotional faces being recognized faster than neutral ones [16,20]. Interestingly, a recent study using the eye-tracking method showed that highly avoidant individuals do not disengage faster from emotional faces [21]. It seems likely that the attentional processes implied in avoidance strategies are not a unitary process, but rather a two-step process consisting of initial vigilance toward threats followed by disengagement and avoidance of attention [22]. According to Chun [23], avoidant individuals exhibit a dual-process response style involving an enhanced, early response to emotional stimuli followed by disengagement and an avoidance of attention at a controlled level of processing. It is believed that this rapid and effortless perception of social–emotional information allows avoidant individuals to react promptly to threats and initiate appropriate avoidant behaviors [24].

The current research is facing a gap concerning the understanding of processes that underlie the attention bias and choice processes related to care-seeking behavior. While attentional biases to emotional stimuli have been demonstrated in avoidant individuals [10,13], little is known about how this process is associated with decision-making; specifically with regard to attachment issues such as choice of care in threatening situations. The relationship between attention and choice during decision-making has been a major focus of research in cognitive science. The information-processing paradigm [25] posits that decision-making is based on fundamental processes of memory, attention, and perception. Researchers have proposed that top-down and bottom-up processes may operate together to link attention and choice [26]. In experimental situations, it has been shown that, while the subject is observing the choice options, information is recovered from memory [27,28]. Decision-relevant memory information from internal values and representations is then used to generate a decision outcome [29,30]. This paradigm may be useful for investigating the decision process in proximity-seeking behavior because it is affected by the individuals’ memorized IWMs. It has been established that eye tracking can provide relevant information about the cognitive processes underlying individuals’ decision-making [31,32]. The Attentional Drift Diffusion model (ADD) states that the alternative, which received the last fixation, coincides with value-based choices [33,34,35]. Recent studies on the attentional bias of attachment styles have begun to successfully use the eye-tracking technique [15]. However, a more reliable inquiry into the attention bias and choice processes related to care-seeking behavior is still lacking. This study therefore attempts to address this research gap. On this basis, we applied the eye-tracking technique to study the attention bias in the choice process of seeking care and the deactivation behavior of avoidant individuals.

### Aims and Hypothesis

Based on these premises, the main aim of this study was to test, using an experimental design with the eye-tracking technique, the propensity to seek care and the role of attention in the choice process under distressed conditions. The secondary aim of this study was to investigate the process of the care-seeking choice during deactivation behavior in avoidant individuals.

The hypotheses are as follows:

(1) We hypothesize that, in general, the last fixation on care and its choice will be the more frequent pattern, and that the exposure to stressful conditions will induce a greater occurrence of this pattern, with respect to neutral conditions;
(2) Regarding the role of avoidance, we hypothesize that there will be no differences in the last fixation on the care pictures between avoidant and low-avoidant individuals. Based on the dual-process model [23], we hypothesize that the attachment deactivation does not happen during the last picture fixation, but rather in the later stages of processing that lead to choice. We expect to find a lower consistency between the last fixation and choice of care, and thus a greater inconsistency in both conditions, i.e., avoidant individuals who last fixate on the care picture will then choose the alternative picture of food more frequently than low-avoidant individuals. Food pictures were selected as the alternative choice because of their enhanced attentional effect [36] and for their strong biological-reward function, irrespective of eating style [37];
(3) With regard to the choice reaction time, we hypothesize that, when the last fixation is a care picture, the choice of care will be faster under threatening conditions. This is because care representation is mostly associated with the proximity-seeking behavior predicted by attachment theory, and thus with fewer elaborate processes;
(4) Finally, we expect thats avoidant individuals will make their care-choice faster under both conditions, given their tendency to divert attention to negative and positive attachment-related information, and overall because of their defenses against the representations of care.

## 2. Materials and Methods

### 2.1. Participants

A total of 72 healthy subjects (27 males and 45 females; mean age 23.9 ± 3.97) participated in the study. None of the participants had a history of and/or current signs/symptoms of neurological and/or psychiatric diseases or a current or recent use of any drugs which could affect cognitive processes. All participants had normal or corrected-to-normal vision.

### 2.2. Measures

#### 2.2.1. Questionnaires

Attachment style was measured using the Italian version of the Experiences in Close Relationships Scale-Revised (ECR-R [38,39]). The questionnaire included 36 relationship-related statements referring to attachment anxiety and avoidance. The anxiety scale included 18 items that reflected an individual’s concerns around rejection and abandonment. The avoidance scale included 18 items assessing discomfort with closeness and dependence. Participants responded on a 7-point Likert scale ranging from 1 (strongly disagree) to 7 (strongly agree). In the current sample, Cronbach alphas were high for the anxiety scale, at *α* = 0.89, as well as for the avoidance scale, at *α* = 0.92. The mean attachment scores were 3.12 (*SD* = 0.97) for attachment anxiety and 2.49 (*SD* = 0.99) for attachment avoidance.

#### 2.2.2. Visual Stimuli

One hundred and eighty images were selected from the following databases to serve as priming or target images:

(a) For emotional stimulation, the International Affective Picture System (IAPS) was used [40]. Forty IAPS pictures were selected for the threatening (e.g., accidents, human attack, dangerous animals), and neutral (e.g., domestic objects) conditions (Appendix A). The contents were associated consistently with higher or lower valence or arousal ratings. Mean ratings of valence and arousal were as per Lang [40] (using a 9-point Likert-like scale: 1 = low; negative). The forty pictures had a mean valence of 2.52 (*SD* = 0.64) and *M* = 5.11, (*SD* = 0.32); and a mean arousal of 6.62 (*SD* = 0.41) and *M* = 2.91 (*SD* = 0.68) for threatening and neutral conditions, respectively. Valence and arousal were significantly different between the two conditions, *F*(1, 38) = 264 and *F*(1, 38) = 438, respectively.
(b) Forty pictures from the The Besançon Affective Picture Set-Adult (BAPS-Adult [41]), depicting comfort-related scenarios where care was represented, were used (Appendix A). Two random lists of twenty pictures were created: one for the threatening and one for the neutral condition. The mean ratings of perceived comfort, valence, and arousal were as per Szymanska [41]). The two lists did not significantly differ on any dimension, with all *F*s < 1.
(c) Forty pictures were taken from Food-Pics_Extended [42]. The pictures were of typical comfort foods (sweet and salty snacks) and were randomly divided into two lists of twenty items: one for the neutral and one for the threatening condition (Appendix A). Mean ratings of calories, palatability, and cravings were as per Blechert [42]. The two lists did not significantly differ on any dimension, with all *F*s < 1.
(d) For the twenty filler trials, sixty neutral pictures were selected from the IAPS and Food-Pics_Extended databases.

### 2.3. Procedure

For the experiment, participants were seated in a comfortable chair in a quiet, light-controlled room at a viewing distance of 70 cm from the screen; a headrest was used to reduce movements during recordings. Visual stimuli were presented using Psychopy3 Software [43] on a 17” LCD monitor (Samsung, 75-Hz refresh rate). Images occupied about 40° of the visual angle horizontally and vertically, and participants were instructed to fixate on the center of the screen. In order to record the picture chosen as well as the reaction times (*RT*, defined as the time elapsed between the stimulus presentation and when the participant completed a key press), subjects were instructed to use a response pad.

**Figure 1:**
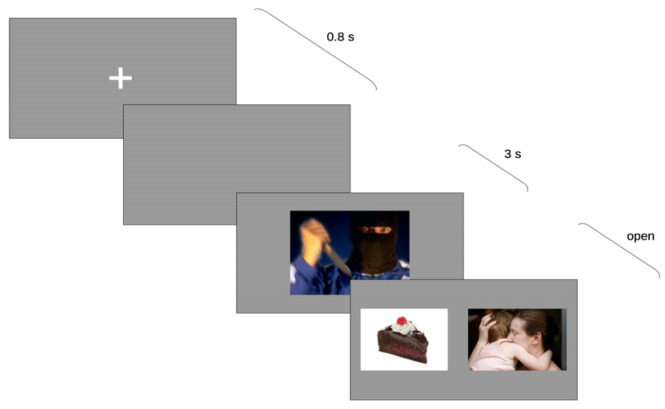
Experimental task used in the study, showing the progression of the visual stimuli in time.

The experiment consisted of sixty randomly intermixed trials: twenty trials with the threatening condition; twenty trials with the neutral condition; and twenty filler trials. Each trial consisted of four isoluminant, sequential images, beginning with a fixation cross in the center of the screen (500 ms), followed by a gray screen (300 ms) and prime picture (3 s), after which two probe pictures were presented side-by-side (Figure 1). Probe pictures remained visible until participants responded. The relative position of the two pictures (left vs. right) was counter-balanced with participants [44]. Under both the neutral and threatening conditions, one of the two pictures depicted a caring scenario whereas the other depicted food. For each trial, the last-fixed image, the image chosen, and the latency of the choice were recorded. In the filler trials, probe pictures had neutral content. Participants filled in the questionnaires and underwent the eye-tracking recording in a counterbalanced way; half started with the questionnaires and half started with the recording.

Prior to the start of the task, participants were adequately informed about the experimental procedure and were allowed to familiarize themselves with it in five practice trials. All the pictures in the practice trials had neutral content and were not included in the experiment. Before beginning the experiment, participants were presented with the following instructions (instructions were presented in Italian, below is the English equivalent):

*“You will see neutral images and other images that will probably make you feel negative emotions, then you will have to choose one of the two images that follow, the one that at that time can help you to overcome the negative emotion of the single image you saw before”.*

#### 2.3.1. Eye Tracking

A mobile, monocular Pupil Labs eye-tracking headset was used to record gaze data, consisting of one 200 Hz eye camera and a 120 Hz world camera to record the visual scene. The eye-tracking headset was connected to a computer (operating system: Ubuntu Linux 20.04 LTS 64-bit, processor type: Intel Core i5-3210 M 2.50 GHz, RAM: 16 GB) via USB-C.

Pupil Capture software (release 3.4) was used to control the calibration and recording. Following the Pupil Labs recommendations for mid-range viewing distances, a 5-point calibration choreography was used to calibrate the eye-tracking device before testing. For this purpose, participants were asked to fixate on a calibration marker that appeared on the screen in a grid-like manner at five locations in the participant’s relevant fields of view. Calibration quality was evaluated through a validation procedure in order to achieve tracking accuracy within the physiological limits (<1 deg visual degrees). During the experiment, the operator used the live video feed of the Pupil Capture software to verify the coherence between the position of the markers on the screen and the calibration grid.

Following data collection, the Pupil Player software (release 3.4) was used to play back eye-tracking recordings to check for quality and define surfaces for data extraction. All the recordings were manually checked by an operator who was blinded to the experimental procedure, and low-quality recordings were excluded from further analysis (n = 6). After extraction using Pupil Player, raw data from single surfaces were further elaborated using a dedicated Python3 script.

#### 2.3.2. Statistical Analysis

By crossing the coupling options (care and food) between the last fixed image and the chosen image, four different patterns were defined: care–consistency; care–inconsistency; food–consistency and food–inconsistency. The different patterns were investigated as emotion regulation processes, both in general and in relation to avoidance. Attachment anxiety was not analyzed because it was not the objective of our study. 

Repeated measures of the ANCOVA were conducted between neutral and threatening conditions, with attachment avoidance entered as a covariate. In all analyses, the covariates were centered around the mean [45]. A contrast analysis between the pattern of care–consistency and the other patterns was performed for each comparison to estimate the differences and measure the statistical significance. The means of the patterns and their RTs were analyzed overall and between the two conditions with attachment avoidance entered as a covariate. In order to highlight possible significant associations, we will show the differences in the last fixation pictures and consistency patterns by using standardized-Z scores (−1 *SD* vs. +1 *SD*) of avoidance.

Other factors that may covary with the choice of food were also taken into consideration: self-reported hunger at the time of the experiment, BMI, and restrained eating, which have been occasionally associated with eating behavior [46,47,48]. Before the ANOVA analyses, a backward stepwise regression between BMI, restrained eating (yes or no), the status of hunger (1 = not at all hungry; 5 = extremely hungry), and the patterns including food pictures was performed. Variables showing no significant association (*p* > 0.05) were not included in further analysis. Statistical analysis was performed with SPSS 20 software (SPSS Inc, Chicago, IL, USA).

## 3. Results

In order to select the factors that may covary with the patterns involving food pictures, a backward stepwise regression was performed. None of those evaluated—BMI (*M* = 21.72; *SD* = 3.22); hunger (*M* = 1.97; *SD* = 0.96); and restrained eating status (no = 62.5%, yes = 37.5%)—were associated with the patterns including food pictures, *p* > 0.05 (Table 1).

The results from the descriptive analysis of data are reported in Table 2. Overall, under threatening and neutral conditions, the care picture was more frequently the last fixated (*M*_Care_ = 22.03, *SD* = 4.69) when compared to the food picture (*M*_Food_ = 17.97, *SD* = 4.69); *F*(1;70) = 13.619, *p* < 0.001, *η*_p_^2^ = 163. No interaction with avoidance emerged; *F*(1;70) = 1.773, *p* > 0.05.

The differences became evident when considering the percentages in the last fixations between care and food under the threatening condition (60.2% care vs. 39.8% food). In fact, while under the neutral condition, the means of the last fixations were almost equal; *F*(1;70) < 1. Under the threatening condition, the difference between care and food was significant (*F*(1;70) = 40.152, *p* < 0.001, *η*_p_^2^ = 365). No interaction with avoidance emerged under the neutral (*F*(1;70) < 1) and threatening conditions (*F*(1;70) = 1.832, *p* > 0.05).

In subsequent analyses, the association between the last-fixated image and the following choice was tested. In 71.33 % of cases, the last image fixated on was coherent with the image chosen; 42.55% of the time concerned the choice of care and 28.78% concerned food. The remaining 28.67% concerned inconsistent choices when fixating on care and choosing food (12.52%), and when fixating on food and then choosing care (16.15%).

Regardless of the conditions, the comparison of the means of the four patterns shows a significant difference (*F*(1;70) = 57.653, *p* < 0.001, *η*_p_^2^ = 0.452), and an interaction effect with avoidance (*F*(1;70) = 6.073, *p* = 0.002, *η*_p_^2^ = 0.080) (Table 3; Total). A contrast analysis showed that the care–consistency pattern was the most frequent. Care–consistency vs. care–inconsistency (*F*(1;70) = 104.397, *p* < 0.001, *η*_p_^2^ = 0.599); care–consistency vs. food–inconsistency (*F*(1;70) = 128.782, *p* < = 0.001, *η*_p_^2^ = 0.648); and care–consistency vs. food–consistency (*F*(1;70) = 18.673, *p* < 0.001, *η*_p_^2^ = 0.211).

The results of the analyses with the RM ANCOVA highlight the significant differences in the four comparisons between the two conditions. Table 3 shows the means and SDs of the four patterns of consistency distributed across the two conditions. With respect to care–consistency, results showed an increase when under the threatening condition (*F*(1;70) = 44.99, *p* < 0.001, *η*_p_^2^ = 391). While the effect of the avoidance interaction was not significant (*F*(1; 70) > 1), its main effect was significant (*F*(1;70) = 10.412, *p* = 0.002, *η*_p_^2^ = 0.129) and found to be associated with both conditions: neutral, with *t* = −2.846, *p* =0.006, and threatening, with *t* = −2.548, *p* = 0.013.

Further, in the care–inconsistency pattern, a decrease in food choice emerged under the threatening condition even when the last fixation was on care (*F*(1; 70) = 22.536, *p* < 0.01, *η*_p_^2^ = 244). The interaction effect with avoidance was not significant (*F*(1; 70) = 1.483, *p* > 0.05). On the other hand, the main effect of avoidance was found to be significant (*F*(1; 70) = 11.472, *p* < 001, *η*_p_^2^ = 141) and was associated with an increase of this pattern in both conditions: neutral, with *t* = 3.475, *p* < 0.001, and threatening, with *t* = 2.450, *p* = 0.017.

In contrast, when considering the food–inconsistency pattern, the choice of care under the threatening condition also increased when food was the last picture fixed upon (*F*(1; 70) = 40.142, *p* < 001, *η*_p_^2^ = 244). The interaction with the avoidance effect was not significant *(F*(1;70) < 1). Finally, in the food–consistency comparison, a decrease of the food choice under the threatening condition was found (*F*(1; 70) = 78.936, *p* < 001, *η*_p_^2^ = 244), and the interaction effect with avoidance was not significant (*F*(1;70) < 1). 

**Figure 2:**
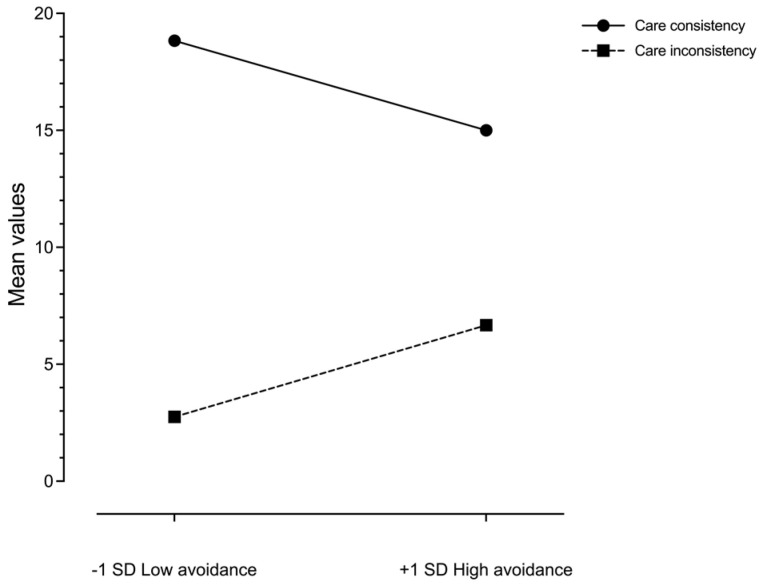
Means of care-consistency and care-inconsistency choices at − 1 *SD* and + 1 *SD* of avoidance scores.

To illustrate the significant effects of avoidance on care–consistency and care–inconsistency patterns of choice, the scores of avoidant individuals were transformed into *Z*-scores (Figure 2). Participants who scored less than one standard deviation (low avoidance) on the avoidance scale, and who more than one standard deviation (high avoidance) were then identified.

Table 4 shows the RTs according to the four patterns under the two conditions. Overall (regardless of the last fixation), our results show that when care is chosen, the RT is shorter under the threatening condition when compared to the neutral condition. In contrast, when food is chosen, the RT increases under the threatening condition when compared to the neutral condition.

**Figure 3:**
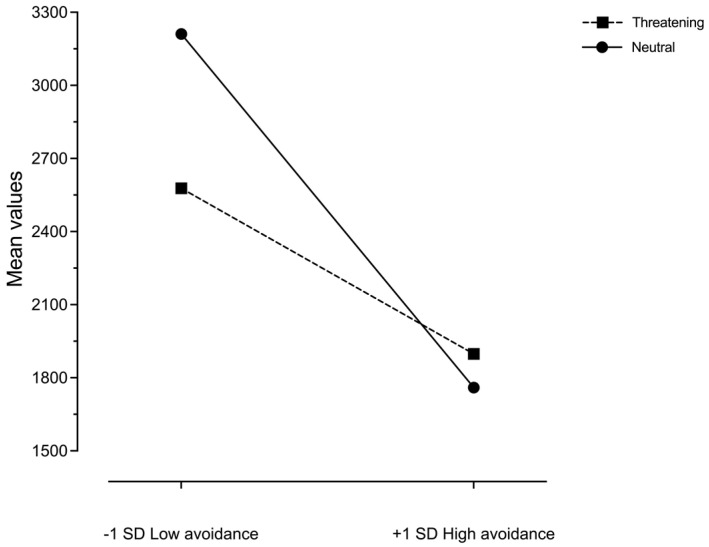
Means of care consistency RTs at −1 *SD* and +1 *SD* of avoidance scores.

The RM ANCOVA results showed a significant difference in three out of four patterns. The comparison on care coherence shows a significant difference between the RTs under the two conditions (*F*(1; 68) = 17.769, *p* < 001, *η*_p_^2^ = 0.207), and an interaction effect with avoidance (*F* (1;68) = 7.130, *p* = 0.009, *η*_p_^2^ = 0.095), which was significant under the neutral conditions (*t*(68) = −2.325, *p* = 0.023) but not under the threatening conditions (*t*(68) = −1.034, *p* > 0.05). In fact, only under the neutral conditions were high avoidance scores associated with a decrease in the RT of care–consistency, compared to low avoidance scores. Figure 3 shows the means of care–consistency RTs for low and high avoidance.

In contrast, there was no significant difference between the two conditions in care–inconsistency (*F*(1; 36) < 1); the interaction effect was also not significant (*F*(1; 36) = 1.601, *p* > 0.05). For food–inconsistency, we found a significant decrease in RTs under the threatening condition (*F*(1; 53) = 6.005, *p* = 0.018. *η*_p_^2^ = 0.102), while the interaction effect was not significant (*F*(1; 36) = 1.601, *p* > 0.05). A significant increase in the threatening condition was also found in the food–consistency (*F*(1; 59) = 6.398, *p* = 0.014. *η*_p_^2^ = 0.098), while the interaction effect was not significant *F*(1; 59) < 1.

## 4. Discussion

The present study was designed to provide a contribution to the understanding of adult avoidant attachment, attention bias, and emotion-regulation patterns. According to attachment theory, we studied the response to the exposure to comfort after distress in general and in avoidant individuals. To this aim, we used an eye-tracking-based protocol to evaluate the choice consistency in the process between the last image fixated on and the image chosen [33]. We hypothesized that care–consistency would be the most frequent pattern, particularly under the threatening condition, while avoidant individuals would demonstrate lower care–consistency, showing that deactivation takes place at the time of choice and not during the last image fixation. RT has been investigated as an index of processing load and engagement, and we expected that, under the threatening condition, the care–consistent choice would be faster when compared to the neutral condition. Finally, we also expected avoidant individuals to perform faster choices in response to care pictures under both conditions.

The large occurrence of last fixations on the care picture and care–consistency is in agreement with our first hypothesis and tends to confirm one of the main postulates of attachment theory: the prioritization of care-seeking when in threatening situations [1]. In fact, under the threatening condition, both care–consistency and food–inconsistency increased, confirming the motivational strength of care representation. This happened not only in the care–consistency pattern, but rather in the food–inconsistency pattern when the last picture fixated was food, and care was the choice. Conversely, the neutral control condition (in the absence of threat) showed a higher frequency of food–consistency and care–inconsistency patterns. Indeed, although the use of food constitutes a strong emotion-regulation strategy [49,50], the representation of care is more effective under threatening situations. The results that emerged from the analysis of the choices are consistent with the normative functioning of the attachment system, and are also in line with our expectations, as are confirmed by the RTs. In fact, under the threatening condition, the short RTs of care-consistent choices indicate spontaneity and less hesitation. Under the threatening condition, care was always chosen faster, whether the last fixed picture was care or food. Conversely, food choice—when under the threatening condition—scored a longer RT not only when it was inconsistent (care vs. food), but even when it was consistent, suggesting greater hesitation and less spontaneity.

In accordance with our second hypothesis, the results indicate specific emotional-regulation strategies related to attachment avoidance. In fact, in avoidant individuals, we found a decrease in care–consistency and an increase in care–inconsistency when compared to individuals with low avoidance. This result was found in avoidant individuals under both conditions, although only the threatening condition generally drove care–consistency. It would therefore seem that, regardless of the experimental condition, these individuals tend to avoid care pictures. This may be interpreted as a general tendency towards attachment deactivation, induced by avoidant defenses, in response to comfort-related attachment pictures [51]. This finding appears particularly relevant because we did not find differences in the last image fixated on in either high- or low-avoidance individuals, but rather the differences arose during the subsequent choice processes. Given that avoidant attachment stems from a history of unsupportive experiences [52], we hypothesize that avoidant individuals elude long-term fixation on care pictures, with their defensive strategies shifting the attention to attachment-unrelated pictures.

In contrast to our expectations, the RT of care–consistency was significantly affected only under the neutral condition, while under the threatening condition high- and low-avoidant individuals did not differ, although they both obtained lower RTs when compared to the neutral condition. In contrast, under the neutral condition—which does not motivate care seeking—only avoidant individuals showed faster RTs. This result requires a different interpretation with respect to the overall result, in which the low RT of care–consistency was found only with the threatening condition. In the total sample, the effect of the threatening condition emerged with a low RT in care–consistency as a normative response of the attachment system to the threat. Conversely, in avoidant individuals, the effect of the care pictures on choice-making appeared more relevant than the effect of the threatening condition itself, as if avoidant individuals wanted to spend as little time as possible in contact with comfort-related attachment images [15]. Another study also showed that insecurely attached individuals reject secure sentences more quickly [53].

In accordance with this concept, we found that avoidant individuals used the deactivation strategy to process comfort-related attachment pictures, suggesting that they considered these stimuli to be threatening. This is in line with attachment theory and other findings [16]. Indeed, as was suggested by some researchers, for avoidant individuals it is the care pictures in particular that are perceived as threatening [14]. This can be interpreted as a way of attentional avoidance of comfort-related attachment stimuli [12,51]. To ensure avoidant individuals’ deactivation, such as shifting their attention away from attachment-related stimuli, automatic brain responses are needed to achieve emotional-stimuli recognition [20]. In this line, other studies have shown that avoidant individuals display increased automatic vigilance toward attachment-related memory recognition. This rapid and effortless recognition could potentially provide an advantage in that it will allow an individual to react immediately to stressful memories, limiting further recall and subsequent negative emotional reactions [24]. In the avoidance dimension, our results show no difference in last fixations but rather in subsequent choices, appearing in line with this process. This is in accordance with the dual process proposed by Chun [23], who posited that defenses are activated following stimulus awareness and not preemptively. Other studies [54] have suggested a binary emotion-regulation strategy employed by avoidant individuals: hyper-vigilance in the initial automatic phase of perception, and an inhibition of emotion in later phases. Overall, our findings suggest that the avoidance of attachment-related pictures may be the product of a defensive strategy leading to the filtering of positive emotional information. This specific deactivation process may stem from the negative representation of others who experienced constant rejection by caregivers during childhood [55]. At the unconscious level, these negative experiences make them think of themselves as unworthy of love, which leads to their early vigilance towards socio-emotional signals [18,24]. These models are thought to persist during development and are then generalized to other individuals outside of primary attachment bonds [56].

In summary, studies within attachment theory have shown that threatening/stressful situations generally motivate individuals to seek care. Conversely, avoidant individuals have developed difficulties in seeking care. Previous studies in this area have shown that avoidant individuals implement attentional disengagement from attachment-related stimuli with a broader attentional field around the attachment figure [51] by fixating later on care images with shorter fixation times [15]. However, a recent study using the eye-tracking method did not confirm these findings [21]. In our study, in addition to the inquiry into eye-fixation dynamics, we included the process of choice. The ADD [33] allowed us to assess gaze and choice as psychobiological parameters of attachment-related emotion regulation. We used this model to investigate the attachment behavior as well as the deactivation process in avoidant individuals. The results showed that the eye-tracking technique, which to the best of our knowledge has never been applied before to the theory of choice using the ADD, can provide a useful method to study the attachment representation of care-seeking propensity. The importance and originality of this study is that it shows the high frequency of care choice as a pattern of consistency between the last-fixated image and the chosen image. Conversely, it shows that the difficulties and defenses of avoidant individuals are expressed with less consistency after fixating on a care image. In this study, the findings should make an important contribution to the field of attachment, particularly on the visual correlation of the care-seeking process and the defenses of avoidant individuals.

### 4.1. Limits and Future Directions

One limit may be related to the choice of comfort food as the only alternative to care pictures. Although they concern two strong forms of reward and emotional regulation, other images of emotional-regulation stimuli will need to be addressed in future studies. Another limitation that may be considered is the use of only the last fixation in the eye-tracking procedure. Several studies indicate that the number and lengths of fixation can also provide a contribution to the understanding of the choice process. However, for our aims, and to identify any inconsistencies in the choices of avoidant individuals, the use of the last fixation seemed more appropriate. Further studies may use the other parameters of the eye tracker to investigate the dimension of attachment anxiety. Similarly, the unconstrained choice between pictures of food and pictures of caring scenarios, although they concern two strong forms of reward and emotional regulation, might be considered as a limit of our study that will need to be addressed in future studies.

### 4.2. Conclusions

Care-seeking in distress situations is one of the cores of attachment theory. This experimental study using eye-tracker methodology investigates this primary motivation in terms of the choice process according to the ADD model, which predicts that the last-fixated image will be the one chosen. The results show that the distress condition significantly increases the consistency of care choice, contrary to the neutral condition. In contrast, avoidant individuals showed a low consistency of choice when fixating on the care pictures in both emotionally neutral and distress conditions. The findings confirm with a novel approach the spontaneity of care-seeking propensity in distress situations. Results also highlight avoidant individuals’ deactivation as a tendency to disengage attention to positive-attachment-related information, and therefore that their defense mechanisms may operate after the fact.

## Figures and Tables

**Table 1:** Correlation matrix of factors that may covary with patterns involving food pictures.

	Patterns of Choice		
Variables	Care-Inconsistency	Food-Inconsistency	Food-Consistency
BMI	0.144	0.153	−0.143
Restrained eating	−0.077	−0.147	0.081
Hunger	0.066	0.031	0.098

**Table 2:** Means, SD, and percentages of the last fixation; picture choices and RTs.

	Neutral			Threatening		
	Care	Food	Diff	Care	Food	Diff.
Last Fixation	9.99 (3.24) (49.95%)	10.01 (3.24) (50.05%)	0.02	12.04 (2.75) (60.2%)	7.96 (2.75) (39.8%)	3.08
Choice	8.93 (4.81) (44.65%)	11.07 (4.81) (55.35%)	2.14	14.54 (4.55) (72.7%)	5.46 (4.55) (27.3%)	9.08
RT Choice (ms)	2890.29 (1820.13)	2488.79 (1432.06)	401.5	2441.09 (1605.88)	2831.88 (1747.63)	−318.41

**Table 3:** Means and SDs of the total four consistency-choice patterns under neutral and threatening conditions.

	Total		Neutral		Threatening		ANCOVA
Patterns	*M*	*SD*	*M*	*SD*	*M*	*SD*	*F*
Care—consistent choice	17.02	6.88	6.82	4.15	10.19	3.93	44.990 ***
Care—inconsistent choice	5.01	4.63	3.17	2.71	1.85	2.49	22.536 ***
Food—inconsistent choice	6.46	4.26	2.11	2.15	4.35	2.98	40.142 ***
Food—consistent choice	11.51	5.65	7.90	3.98	3.61	2.92	78.396 ***
Total	40.00		20.00		20.00		

*** *p* < 0.001.

**Table 4:** RT (ms) means of the four consistency choice patterns under neutral and threatening conditions.

	Neutral		Threatening		ANCOVA
Patterns	*M*	*SD*	*M*	*SD*	*F*
RT Care—consistent choice	2787	1641	2438	1552	17.769 ***
RT Care—inconsistent choice	2401	1677	2657	2309	<1
RT Food—inconsistent choice	2552	2000	2233	1791	6.005 *
RT Food—consistent choice	2439	1377	2830	1694	6.398 *
Total	2602	1502	2518	1595	

*** *p* < 0.001; * *p* < 0.05.

## Data Availability

The data presented in this study are available upon request from the corresponding author.

## Appendix A

(IAPS)

Threatening prime: 1120—1300—1304—1930—3230—3500—3530—6250—6312—6313—6315—6350—6370—6510—6540—6550—6560—6570—9414—9635.1.

Neutral prime: 7040—7002—7004—7018—7020—7026—7053—7059—7061—7062—7080—7090—7095—7150—7175—7185—7205—7211—7233—7235.

(BAPS)

Care pictures: 1—2—3—4—5—8—9—11—12—14—15—16—19—20—22—23—24—25—26—27—28—29—30—32—34—35—40—42—46—47—48—49—50—51—52—55—56—57—59—64.

(Food-Pics)

Food pictures: 4—15—26—31—43—44—48—66—67—80—93—94—102—103—104—107—109—113—115—116—127—133—137—140—150—170—173—177—183—186—205—225—286—287—510—539—673—676—869—878.
